# MRI features predictive of negative surgical margins in patients with HER2 overexpressing breast cancer undergoing breast conservation

**DOI:** 10.1038/s41598-017-18758-0

**Published:** 2018-01-10

**Authors:** Brittany Z. Dashevsky, Jung Hun Oh, Aditya P. Apte, Blanca Bernard-Davila, Elizabeth A. Morris, Joseph O. Deasy, Elizabeth J. Sutton

**Affiliations:** 1 Department of Radiology & Biomedical Imaging, University of California, San Francisco, CA USA; 2 Department of Radiology, Memorial Sloan Kettering Cancer Center, New York, NY USA; 3 Department of Medical Physics, Memorial Sloan Kettering Cancer Center, New York, NY USA

## Abstract

Here we develop a tool to predict resectability of HER2+ breast cancer at breast conservation surgery (BCS) utilizing features identified on preoperative breast MRI. We identified patients with HER2+ breast cancer who obtained pre-operative breast MRI and underwent BCS between 2002–2013. From the contoured tumor on pre-operative MRI, shape, histogram, and co-occurrence and size zone matrix texture features were extracted. In univariate analysis, Spearman’s correlation coefficient (Rs) was used to assess the correlation between each image feature and an endpoint (surgical re-excision). For multivariate modeling, we employed a support vector machine (SVM) method in a manner of leave-one-out cross-validation (LOOCV). Of 109 patients with HER2+breast cancer who underwent BCS, 39% underwent surgical re-excision. 62% had residual cancer at re-excision. In univariate analysis, solidity (Rs = −0.32, p = 0.009) and extent (Rs = −0.29, p = 0.019) were significantly associated with re-excision. Skewness in post-contrast 1, 2, and 3 (Rs = 0.25, p = 0.045; Rs = 0.30, p = 0.015; Rs = 0.28, p = 0.026) and kurtosis in post-contrast 1 (Rs = 0.26, p = 0.035) were also statistically significant. LOOCV-based SVM test achieved 74.4% specificity and 71.4% sensitivity when 21 features were used. Thus, tumor texture, histogram and morphological MRI features may assist surgical planning, encouraging wide margins or mastectomy in patients who may otherwise go on to re-excision.

## Introduction

For patients with early stage breast cancer, there is no significant difference in disease-free survival for patients receiving breast conserving surgery (BCS) in comparison to mastectomy^1–3^. Thus breast conservation is now the standard of care for early stage tumors. Tumor at the surgical margin is the strongest predictor of locoregional recurrence^4,5^, resulting in a two-fold increased risk of recurrence. This effect persists despite adjuvant treatment with chemotherapy, radiation therapy or hormonal therapy^6^. Thus, the American Society of Clinical Oncology recommends that surgically inked margins from BCS be negative for malignancy^6–8^.

Currently, 17 to 25% of women require re-excision after BCS to obtain negative surgical margins^7,9^. This rate of re-excision is increased in certain molecular subtypes, such as HER2 overexpressing (HER2+) breast cancer, with an odds ratio of 2.0 when compared to HER2 negative breast cancers^10^. Re-excision is associated with increased health care costs and can result in increased morbidity, patient anxiety and poor cosmesis.

MRI is the most sensitive imaging modality for defining breast cancer extent^11–13^. Studies evaluating the effect of pre-operative MRI on re-excision rates have had mixed results, with some studies demonstrating decreased re-excision rates^14^ and others demonstrating no significant difference^15^. Currently the MRI Breast Imaging Reporting And Data System (BIRADS) lexicon is used to characterize the index cancer and extent of disease^16,17^. On pre-operative MRI, HER2+ cancers demonstrate higher rates of multicentric/multifocal disease^13^, skin-nipple-periareolar involvement^18^ and increased rate of rapid early contrast uptake^19^ compared to other molecular subtypes.

For these more complex tumors, image analysis with advanced computer algorithms may assist the radiologist in better delineating tumor extent and quantifying predictive image features. Computational software has been developed for this purpose. From a segmented three dimensional volume of a tumor, computational software can evaluate the tumor’s texture^20^. Texture features can be computed from the grey-level co-occurrence matrix (GLCM)^20^ and the grey-level size zone matrix (GLSZM)^21^ calculated within the 3D volume. Texture features often include 4 GLCM-based features (energy, entropy, homogeneity, and contrast) and 11 GLSZM-based features. Voxel gray scale can be represented as a histogram, allowing determination of histogram features such as variance, skewness and kurtosis^22^. Lastly, shape-based morphologic features can be determined from the segmented volume. These features have already been used to differentiate benign and malignant disease^23^ and breast cancer molecular subtypes^24^ and to correlate MR imaging features of invasive ductal breast cancer with the OncotypeDx recurrence score which predicts patient prognosis^25^.

Since patients often undergo preoperative MRI and yet many surgical specimens have positive surgical margins, we hypothesize that additional features of the entire tumor (within the confines of the margin, as well as the margin) may be identified with quantitative imaging and these computational features on preoperative MRI may be used to predict negative pathologic tumor margins at initial BCS, conferring tumor resectability.

## Results

One hundred and nine women with HER2+ invasive breast cancer (100 invasive ductal carcinoma (IDC); nine mixed invasive ductal and lobular (IMC)) who had a preoperative breast MRI underwent BCS with a mean age of 48.4 years (range 27–79). Preoperative MRIs were performed at 3 Tesla (23 patients) or 1.5 Tesla (86 patients), depending on scanner availability. Following initial BCS 42/109 (39%) patients underwent surgical re-excision and 67/109 (61%) did not (Table 1).

**Table 1:** Patient characteristics.

	Patients who did not require re-excision N = 67 (61%)	Patients who underwent re-excision N = 42 (39%)	p-value
Age range (years)	48 (30–79)	49 (27–74)	0.22
Breast Density			0.26
1	0 (0%)	1 (2%)	
2	5 (7%)	6 (14%)	
3	40 (60%)	28 (67%)	
4	18 (27%)	7 (17%)	
NA	4 (6%)	0 (0%)	
Localization			**<0.01**
No	**26 (39%)**	**5 (12%)**	
Yes	**41 (61%)**	**37 (88%)**	
Localization method			0.92
Wire	39 (58%)	35 (83%)	
Seed	2 (3%)	2 (5%)	
NA	26 (39%)	5 (12%)	
Bracket			0.56
No	39 (58%)	34 (81%)	
Yes	2 (3%)	3 (7%)	
NA	26 (39%)	5 (12%)	
Type of invasive cancer			0.07
IMC	3 (4%)	6 (14%)	
IC	64 (96%)	36 (86%)	
ER			**0.01**
Negative	**17 (25%)**	**21 (50%)**	
Positive	**49 (73%)**	**21 (50%)**	
Borderline	**1 (1%)**	**0 (0%)**	
PR			**0.01**
Negative	**26 (39%)**	**27 (64%)**	
Positive	**40 (60%)**	**15 (36%)**	
Borderline	**1 (1%)**	**0 (0%)**	
DCIS			**0.01**
No	**59 (88%)**	**29 (69%)**	
Yes	**8 (12%)**	**13 (31%)**	
Poorly differentiated tumors	60 (90%)	38 (90%)	0.26
Positive lymph nodes	**26 (39%)**	**12 (29%)**	**<0.01**
Size on MRI (cm)	**2.0 ± 0.8**	**2.6 ± 1.7**	**0.02**
Largest dimension of excised tissue (cm)	5.8 ± 3.0	5.6 ± 2.8	0.98
Size of tumor on pathology	1.7 ± 0.8	1.3 ± 0.9	0.11
Multifocal/Multicentric on MRI	**16 (24%)**	**17 (40%)**	**<0.01**

M: Mixed invasive lobular and invasive ductal carcinoma.

D: Invasive ductal carcinoma.

DCIS: Ductal carcinoma *in situ*.

Bracket: Indicates 2 wires or 2 seeds were utilized to delineate extent of disease prior to surgery.

There was no significant difference in breast density for patients who went on to re-excision, as compared to those who did not (p = 0.26), with breast density considered heterogeneously dense or extremely dense for 84% of patients who went on to re-excision, as compared to 87% of patients who did not. Among patients who went on to re-excision after BCS, 88% had pre-operative image guided localization of the breast cancer, compared to 61% of patients who did not (p < 0.01). While there was a significant difference in whether preoperative image-guided localization was performed, there was no significant difference in the method of image-guided localization (radioactive seed or wire) (p = 0.92), or whether more than one seed or wire was used to bracket the cancer during localization (p = 0.56). Cancers that went on to re-excision were more often associated with ductal carcinoma *in situ* (DCIS) (31 vs. 12%; p = 0.01), were less often estrogen receptor positive (ER+) (50% vs. 73%; p = 0.01) and less often progesterone receptor positive (PR+) (36% vs. 60%; p = 0.01), as compared to cancers that did not undergo re-excision (Table 1).

Of the 42 patients that underwent re-excision, 25/42 (59.5%) had tumor on the inked margin of the resected specimen, 13/42 (31.0%) had tumor within 1 mm of the margin and 4/42 (9.5%) were re-excised for other reasons (residual calcifications (1/4), discordant pathology (1/4) or tumor within ≥2 mm of the surgical margin (2/4)) (Table 2). There was no significant difference in residual carcinoma on re-excision when comparing patients who underwent re-excision for ink on tumor (17/25 vs 9/13) and those who underwent re-excision for tumor within less than 1 mm of the surgical margin. In contrast, patients who underwent surgical re-excision for other reasons (4/42) did not have residual tumor (Table 2).

**Table 2:** Reason for re-excision.

Reason for Re-excision	Residual Carcinoma on Re-excision	Mastectomy on First Re-excision
Ink on Tumor (N = 25)	17 (68%)	11 (44%)
<1 mm Margin (N = 13)	9 (69%)	4 (31%)
Other (N = 4)	0 (0%)	0 (0%)
Total (N = 42)	26 (62%)	15 (36%)
p-value*	**<0.01**	**0.03**

*Among all three reasons for re-excision, p-value of <0.05 reflects a significant difference in residual carcinoma and the number of patients who had mastectomy on first re-excision.

While the maximum dimension of the primary tumor on MRI was greater for patients who required re-excision (2.6 ± 1.7 cm) compared to those who did not (2.0 ± 0.8 cm; p = 0.02), there was no significant difference in the maximum size of the tumor at pathology (1.4 ± 0.9 cm vs 1.7 ± 0.8 cm) as shown in Table 3. Among patients requiring one or ≥ two re-excisions there was also no significant difference in MRI size (p = 0.87) or size on pathology (p = 0.65), as shown in Table 3. Forty percent of patients who underwent re-excision had multifocal/multicentric disease on MRI, compared to 24% of those who did not (p < 0.01).

**Table 3:** Comparison of tumor on MRI and pathology.

	Multifocal/ Multicentric	Size on MRI	Size on pathology
No re-excision (N = 67)	16 (24%)	2.0 ± 0.8	1.7 ± 0.8
One re-excision (N = 31)	17 (55%)	2.6 ± 1.7	1.3 ± 0.9
≥2 re-excisions (N = 11)	1 (9%)	2.5 ± 1.9	1.5 ± 0.9
p-value*	**<0.01**	**0.02**	0.11

*Among tumors that underwent no, one, and ≥two re-excisions, p-value of <0.05 reflects a significant difference in multifocal/multicentric disease and tumor size on MRI.

**Figure 1:**
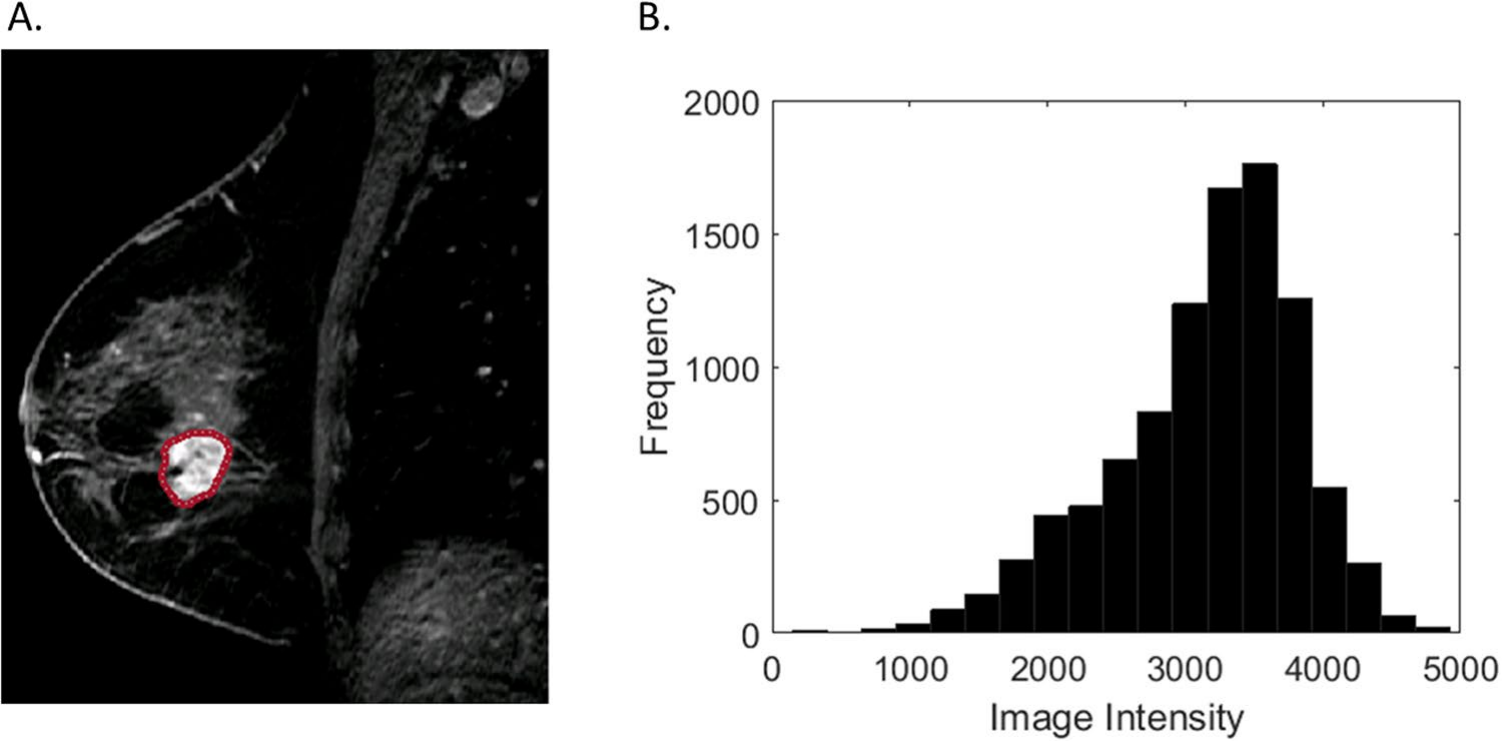
Representative HER2+ breast cancer with negative margins at initial BCS on T1- weighted fat-suppressed first post-contrast images (**A**): solidity = 0.94, extent = 0.74. Histogram plot (**B**) of the intensity values from the region of interest highlighted in (**A**).

**Figure 2:**
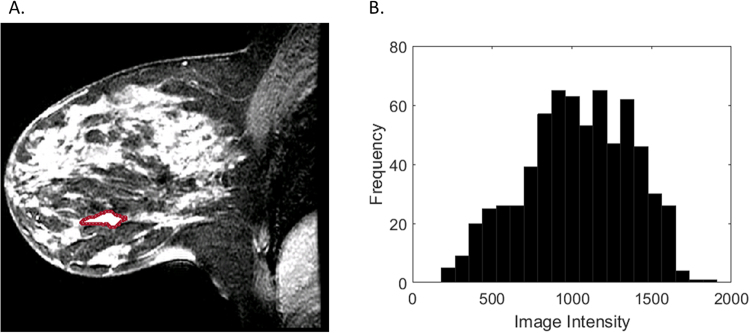
Representative HER2+ breast cancer requiring one re-excision on T1-weighted fat-suppressed first post-contrast images (**A**): solidity = 0.87, extent = 0.66. Histogram plot (**B**) of the intensity values from the region of interest highlighted in (**A**).

**Figure 3:**
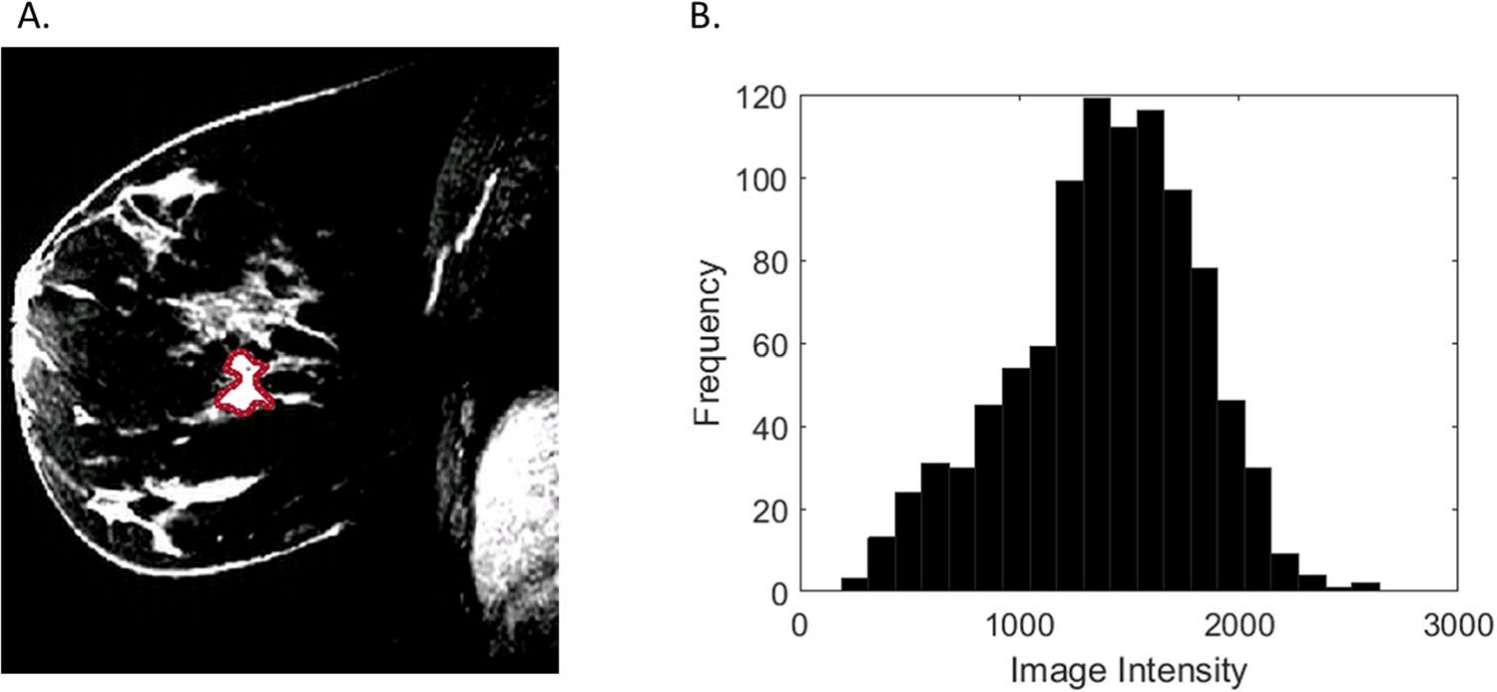
Representative HER2+ breast cancer requiring two re-excisions on T1-weighted fat-suppressed first post-contrast images (**A**): solidity = 0.80, extent = 0.54. Histogram plot (**B**) of the intensity values from the region of interest highlighted in (**A**).

**Figure 4:**
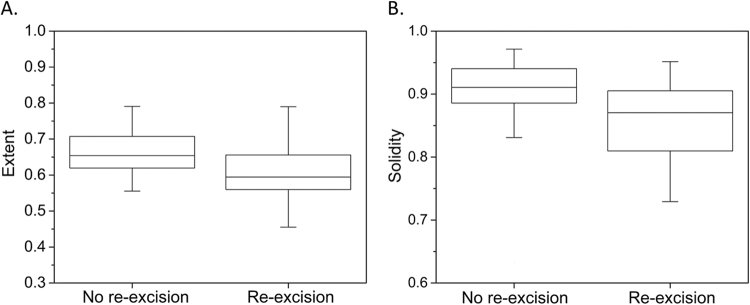
Comparison of no re-excision group with re-excision group for (**A**) extent and (**B**) solidity.

**Figure 5:**
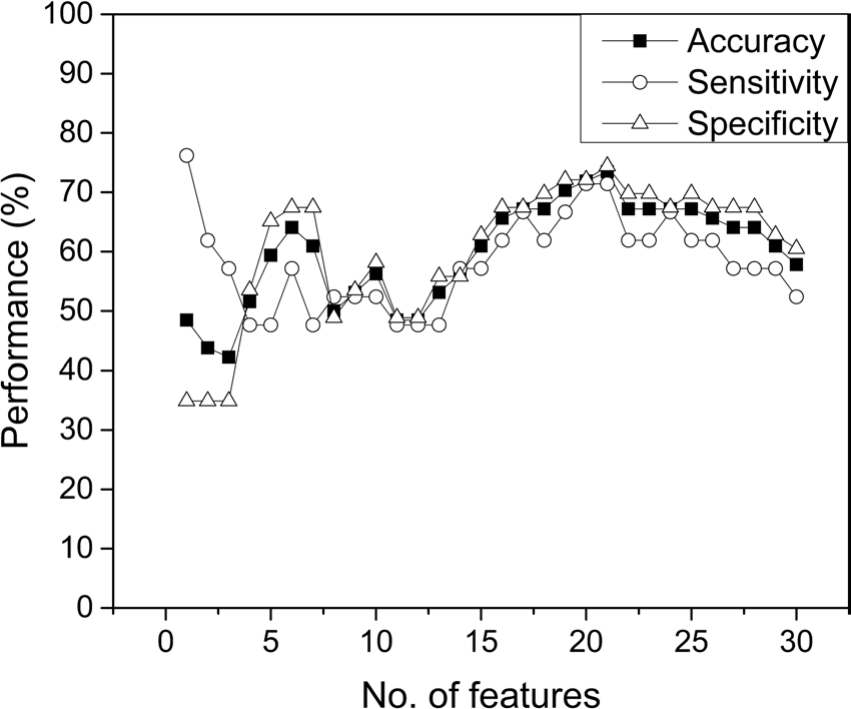
Accuracy, sensitivity, and specificity of SVM models obtained with an increasing number of the top ranked features.

Representative post-contrast MR images of HER2+ breast cancers requiring no re-excision, one re-excision, and two re-excisions are shown in Figs 1–3. In univariate analysis, solidity (Rs = −0.32, p = 0.009) and extent (Rs = −0.29, p = 0.019) were significantly associated with re-excision. Figure 4 shows boxplots depicting significant difference between patients with no re-excision and re-excision with p = 0.044 and p = 0.029 for extent and solidity, respectively, using the two-sample t-test. Skewness in post-contrast 1, 2, and 3 showed statistically significant correlation with re-excision (Rs = 0.25, p = 0.045; Rs = 0.30, p = 0.015; Rs = 0.28, p = 0.026). Kurtosis in post-contrast 1 was also statistically significant with Rs = 0.26 (p = 0.035). Figure 5 shows the change of accuracy, sensitivity, and specificity of SVM models obtained with an increasing number of the top ranked features. The features were ranked separately on each LOOCV dataset using a BW-ratio test. LOOCV-based SVM test achieved74.4% (95% confidence interval [CI]: 59.8–85.1) specificity, and 71.4% (95% CI: 50.0–86.2) sensitivity when the top 21 features were used. Table 4 shows the 21 image features. Interestingly, skewness for all 4 scans was selected in the SVM model. While only one GLCM-based texture feature was selected, 9 GLSZM-based texture features were used in the SVM model.

**Table 4:** Top 21 image features that were used in the best support vector machine model.

Feature type	Scan	Feature	Ranking
	Pre-contrast	Skewness	10
Histogram	Post-contrast 1	Skewness	2
	Post-contrast 2	Skewness	1
	Post-contrast 3	Skewness	3
	Post-contrast 1	Kurtosis	5
	Post-contrast 3	Kurtosis	13
	Pre-contrast	Minimum intensity	8
	Post-contrast 1	Median intensity	17
Shape	Pre-contrast	Solidity	4
	Pre-contrast	Extent	6
	Pre-contrast	Eccentricity	7
GLCM	Post-contrast 1	Contrast	9
GLSZM	Pre-contrast	Large-zone emphasis (LZE)	18
	Post-contrast 2	Large-zone emphasis (LZE)	14
	Post-contrast 3	Large-zone emphasis (LZE)	15
	Post-contrast 1	Low-intensity zone emphasis (LIZE)	21
	Post-contrast 2	Low-intensity zone emphasis (LIZE)	19
	Post-contrast 1	High-intensity zone emphasis (HIZE)	11
	Post-contrast 2	Low-intensity large-zone emphasis (LILZE)	12
	Post-contrast 3	Low-intensity large-zone emphasis (LILZE)	16
	Post-contrast 2	High-intensity large-zone emphasis (HILZE)	20

## Discussion

Despite advances in breast cancer imaging, determining which cancers are amenable to BCS is challenging, with re-excision not uncommon. Cancers of patients who underwent re-excision were more often multifocal, larger on MRI, had associated DCIS and were less often ER+/PR+. Patients who went on to re-excision were significantly more likely to undergo image-guided pre-operative localization, as compared to those who did not. This may in part be attributed to larger tumor size on MRI and increased multifocal/multicentric disease.

Tumors requiring re-excision were significantly larger on MRI but not on pathology, as compared to tumors successfully excised at initial BCS. Discrepancy between MRI and pathology tumor measurements has been reported in prior studies^11–13^. MRI measurements are made when the breast is perfused with contrast, while this is not the case for pathology measurements where the tissue is fixed in formalin. Notably, the discrepancy in MRI tumor measurement, with respect to pathology, was significantly greater for lesions that went on to re-excision.

Since preoperative MRI is often used to plan surgical resection, we used computational analysis to evaluate additional quantitative features which may identify patients at high risk of re-excision. Computer-aided diagnosis (CAD) has become increasingly sophisticated. While initial studies sought to distinguish benign and malignant lesions, CAD systems can now identify more subtle differences between breast cancer molecular subtypes^27–29^. Recent studies have correlated quantitative MR features, similar to those used here, with outcome measures such as tumor response to neoadjuvant chemotherapy^30–32^ and risk of tumor recurrence as predicted by genomic assays^25,33^. However, this is the first study to correlate quantitative MR imaging features with surgical outcome. We evaluated features that contribute to the internal tumor signature (histogram and texture), as well as features which define the tumor margin/shape (solidity, extent, and eccentricity).

Using computational analysis we evaluated imaging features not readily apparent by the interpreting radiologist and demonstrated that these features are valuable in terms of predicting the success of initial BCS and are correlated with patient prognosis. Our machine-learning-based predictive model, using the top 21 imaging features identified on preoperative MRI, can predict HER2+ tumors that were successfully excised at initial BCS from those requiring ≥1 re-excision, with 74.4% specificity and 71.4% sensitivity. Among the top 21 imaging features, 1 GLCM and 9 GLSZM matrix features were included, with skewness being the highest rated feature. Skewness and kurtosis indicate the degree of asymmetry and flatness of the intensity histogram, respectively. Our results showed that a less left-skewed and a more peaked intensity distribution appear to be associated with the risk of re-excision. Prior studies have suggested that kurtosis is associated with lesion neovascularity. Studies by Ashraf *et al*. 2013, and Sutton *et al*. 2015, found that increased kurtosis was associated with more aggressive estrogen receptor positive breast cancers that had increased risk of tumor recurrence^25,33^. Though here we are evaluating a different subtype of breast cancer, HER2+ tumors, we find that skewness and kurtosis are associated with tumors that are more difficult to manage, here in terms of resectability at BCS.

Two shape-based features, solidity and extent, also showed statistical significance in univariate analysis in differentiating tumors that went on to successful initial BCS as compared to those requiring re-excision. Solidity is a measurement of the convexity of a lesion, as described by El Naqa *et al*.^34^. Extent describes the proportion of pixels in the smallest bounding rectangular box that contains the lesion. This means that lesions that are irregularly shaped, with less convex margins are less likely to undergo successful initial BCS. While tumor shape is known to be an important feature in predicting tumor behavior and shape-descriptors are included in the BIRADS lexicon, CAD may more precisely quantify these features.

In this study 38.5% of patients underwent re-excision. This reflects the re-excision rate among this particular cohort of HER2+ patients who received pre-operative MRI, at a major cancer center with dedicated breast surgeons. Our high re-excision rate likely reflects both the aggressiveness of this breast cancer subtype while trying to obtain optimal cosmesis, thereby underscoring the need to evaluate this subpopulation of breast cancer. As there are no national guidelines, nor practice benchmark, it is hard to know what the national norm would be. While current published BCS re-excision rates range from 17 to 25%, there is up to double the frequency of re-excision for positive margins in patients with more aggressive breast cancer subtypes, such as HER2+^7–10^.

We found 55% of patients that required one re-excision had multifocal or multicentric disease, compared to only 24% of patients who had successful initial BCS. This is congruent with published studies demonstrating decreased success of BCS in patients with multifocal or multicentric disease. Yet, among patients requiring ≥2 re-excisions, only 9.1% of patients had multifocal or multicentric disease. This can be attributed to a high rate of mastectomy at first re-excision in patients with multifocal/multicentric disease. At initial re-excision, 15 patients received total mastectomy. We cannot account for those patients who would have required additional re-excisions had a more conservative approach been taken at initial re-excision, thus dampening differences between those requiring one re-excision and ≥2 re-excisions.

Furthermore, results were determined from a retrospective review and while the documented reason for re-excision is stated, surgical management of breast lesions is a complex decision, taking into account a multitude of factors which we are unable to account for. Since commencing this study recommendations for re-excision have changed, such that re-excision is now only recommended for patients with ink on tumor at BCS. We found no significant difference in rates of residual carcinoma when comparing patients with ink on tumor to patients with margins of less than 1 mm, thus justifying inclusion of patients with tumor margins of less than 1 mm in this study. We targeted a specific molecular subtype of invasive breast cancer (HER2+) for analysis due to their higher rate of re-excision and in order to obtain a more uniform set of cancers. It is likely that the meaning of these image features can be extended to other molecular subtypes, but further analysis is needed. At the time of this study, the clinical breast imaging protocol included high spatial and low temporal resolution imaging. Literature suggests high temporal resolution and multiparametric MRI may provide additional promising imaging information in future^35^.

In conclusion, utilizing our machine learning model to evaluate HER2+ breast cancers on preoperative MRI, based on the top 21 image features, we were able to depict 71.4% sensitivity and 74.4%. We found that image features including morphological features, skewness, kurtosis, and texture features are associated with re-excision post BCS in patients with HER2+ breast cancer, and thus these features may be considered during surgical planning in order to decrease the rate of women requiring re-excision.

## Methods

This Health Insurance Portability and Accountability Act compliant retrospective study received institutional review board approval and a waiver of informed consent.

### Patient Cohort

Utilizing a retrospective search of our electronic hospital information system, we identified breast cancer patients who underwent breast conservation surgery between 2002 and 2013, with the following additional inclusion criteria: (a) HER2+ invasive breast cancer; (b) pre-operative bilateral breast MRI and (c) BCS with pathologic evaluation of the excised specimen. Exclusion criteria included patients who: (a) received neoadjuvant chemotherapy; (b) had tumors with marked clip artifact and (c) had a personal history of treated breast cancer. 109 women met these criteria.

### MR Image Acquisition

Imaging was performed on either 1.5- or 3.0-T whole-body MRI unit (GE Medical Systems, Waukesha, WI) equipped with a dedicated 8- or 16- channel surface breast coil. For all patients, sagittal T1-weighted fat-suppressed sequences were obtained prior to administration of intravenous gadopentetate dimeglumine. Following contrast administration (0.1 mmol/kg body weight at 2 ml/sec with an automatic injector), three consecutive T1-weighted fat suppressed sequences were acquired, with the following imaging parameters: repetition time 7.4msec; echo time 4.2msec; flip angle 10°; bandwidth 32 kHz; field of view 18–22 cm; acquisition matrix 256 × 192; NEX 1; slice thickness 3 mm; gap 0 mm; temporal resolution 90 s.

### Computer Based Image Analysis

Methods are similar to those previously employed by Sutton *et al*.^25^. One radiologist with four years of experience, blinded to patient outcome, contoured the tumor boundary by hand on the fatsuppressed T1weighted first postcontrast images. The tumor boundary was then mapped to the pre-contrast and two additional post-contrast T1 sequences. Shape-based, histogram-based, GLCM and GLSZM matrix texture features were extracted from the segmented tumor on the pre and three post-contrast T1-weighted fat-suppressed sequences using our in-house software (Computational Environment for Radiotherapy Research)^26^. For extracting texture features, intensity values were quantized into 16 levels.

### Statistical Analysis

Descriptive statistics were generated and associations were examined using univariate analysis. Comparisons between groups were assessed using χ^2^ or Fisher’s exact test for categorical variables and t-test for continuous variables. Spearman’s correlation coefficient (Rs) was used to assess the correlation between each image feature and an endpoint (surgical re-excision). A two-sample t-test was performed for image features to investigate whether there is a significant difference between two groups (no re-excision group vs re-excision group). Statistical significance was determined with p < 0.05.

For multivariate modeling, we employed a support vector machine (SVM) in a manner of leave-one-out cross-validation (LOOCV). At each iteration of LOOCV, for feature selection, the ratio of between-group to within-group sums of squares (BW-ratio) test was used^25^. Using the sorted features based on their BW-ratios, non-linear SVM classifiers with radial basis kernel were constructed with multiple features in a way that 1, 2, 3, and so forth up to the top 30 features were used. For training SVM classifiers, two parameters were required: soft margin parameter = 1000 that controls the penalty of classification error and gamma = 0.005 which controls the degrees of non-linearity. These two values were chosen after several tests with different combinations. Sensitivity is defined as the percentage of surgical re-excision patients who are correctly classified. Specificity is defined as the percentage of non-surgical re-excision patients who are correctly classified.

### Clinical Data/Breast Conservation Surgery

Clinical data collected included patient’s age, diagnosis, MRI and pathology features, pre-operative localization, tumor margin status at pathology, reason for re-excision, and pathology results from any additional re-excisions of the same tumor. Fifty-six percent (61/109) of patients presented with a palpable mass. Patients underwent pre-operative wire or radioactive seed localization for non-palpable tumors. At our institution BCS is performed in patients with early stage disease. The precise treatment plan is based upon suggestions by a multidisciplinary team, which takes into account patient risk factors for recurrence, such as family history of breast cancer and prior radiation therapy. Due to the high volume of cases at our institution there are several breast surgeons. Our data includes BCS performed by 12 different attending breast surgeons. Re-excision was routinely performed when pathology identified tumor within 1 mm of the surgical margin. Exceptions are noted in the results section.
